# Association of rheumatoid arthritis with chronic kidney disease

**DOI:** 10.1093/ckj/sfaf391

**Published:** 2025-12-12

**Authors:** Mathias Ausserwinkler, Andreas Kronbichler, Sophie Gensluckner, Simon Aberger, Bernhard Paulweber, Eugen Trinka, Patrick Langthaler, Bernhard Iglseder, Maria Flamm, Elmar Aigner, Bernhard Wernly

**Affiliations:** Department of Internal Medicine, Elisabethinen Hospital Klagenfurt, Austria; First Department of Medicine, Landeskrankenhaus – Uniklinikum Paracelsus Medical University Salzburg, Austria; Department of Internal Medicine IV, Medical University, Innsbruck, Austria; Department of Medicine, University of Cambridge, Cambridge, UK; First Department of Medicine, Landeskrankenhaus – Uniklinikum Paracelsus Medical University Salzburg, Austria; First Department of Medicine, Landeskrankenhaus – Uniklinikum Paracelsus Medical University Salzburg, Austria; First Department of Medicine, Landeskrankenhaus – Uniklinikum Paracelsus Medical University Salzburg, Austria; Department of Neurology, Neurointensive Care and Neurorehabilitation, Member of the European Reference Network EpiCARE, Christian Doppler University Hospital, Centre for Cognitive Neuroscience, Paracelsus Medical University Salzburg, Austria; Neuroscience Institute, Christian Doppler University Hospital, Centre for Cognitive Neuroscience, Paracelsus Medical University Salzburg, Salzburg, Austria; Department of Neurology, Neurointensive Care and Neurorehabilitation, Member of the European Reference Network EpiCARE, Christian Doppler University Hospital, Centre for Cognitive Neuroscience, Paracelsus Medical University Salzburg, Austria; Neuroscience Institute, Christian Doppler University Hospital, Centre for Cognitive Neuroscience, Paracelsus Medical University Salzburg, Salzburg, Austria; Department of Geriatric Medicine, Christian Doppler University Hospital, Paracelsus Medical University Salzburg, Salzburg, Austria; Institute of General Practice, Family Medicine and Preventive Medicine, Center for Public Health and Healthcare Research, Paracelsus Medical University Salzburg, Salzburg, Austria; First Department of Medicine, Landeskrankenhaus – Uniklinikum Paracelsus Medical University Salzburg, Austria; First Department of Medicine, Landeskrankenhaus – Uniklinikum Paracelsus Medical University Salzburg, Austria; Institute of General Practice, Family Medicine and Preventive Medicine, Center for Public Health and Healthcare Research, Paracelsus Medical University Salzburg, Salzburg, Austria

**Keywords:** chronic inflammation, CKD, CRP, proteinuria, quality of life

## Abstract

**Background:**

Rheumatoid arthritis (RA) has been linked to an increased risk of chronic kidney disease (CKD), but the predominant renal phenotype and its independence from established risk factors remain unclear. We examined the RA–CKD association in a large, population-based cohort.

**Methods:**

RA and CKD were defined using American College of Rheumatology/European Alliance of Associations for Rheumatology and KDIGO criteria. Logistic regression models were employed with stepwise adjustment: first for cardiovascular risk [Systematic COronary Risk Evaluation 2 (SCORE2)], followed by a model including age, sex, metabolic syndrome, smoking status, non-steroidal anti-inflammatory drug (NSAID) use and high-sensitivity C-reactive protein. Interaction terms were tested to evaluate effect modification.

**Results:**

Among 9665 participants from the Paracelsus 10,000 cohort, 296 (3.1%) had RA. CKD prevalence was higher in the RA group compared with controls (11.8% vs 6.7%, *P* < .001). Albuminuria at preserved estimated glomerular filtration rate was the dominant renal manifestation in RA (6.8% vs 4.2%, *P* = .027). In unadjusted analyses, RA was associated with higher odds of CKD [odds ratio (OR) 1.86, 95% confidence interval (CI) 1.30–2.68], an association that persisted after cardiovascular risk adjustment. However, the association was attenuated and no longer statistically significant in the fully adjusted model (OR 1.43, 95% CI 0.96–2.13). A significant interaction was observed with NSAID use (*P* = .042), whereby the association was largely confined to RA patients not using NSAIDs.

**Conclusions:**

RA is associated with a higher prevalence of CKD, primarily driven by albuminuria at preserved kidney function. This distinct renal phenotype appears largely mediated by metabolic comorbidities rather than inflammation alone. Our findings highlight the need for systematic albuminuria screening in RA patients to enable earlier detection and intervention.

## GRAPHICAL ABSTRACT

**GRAPHICAL ABSTRACT:**
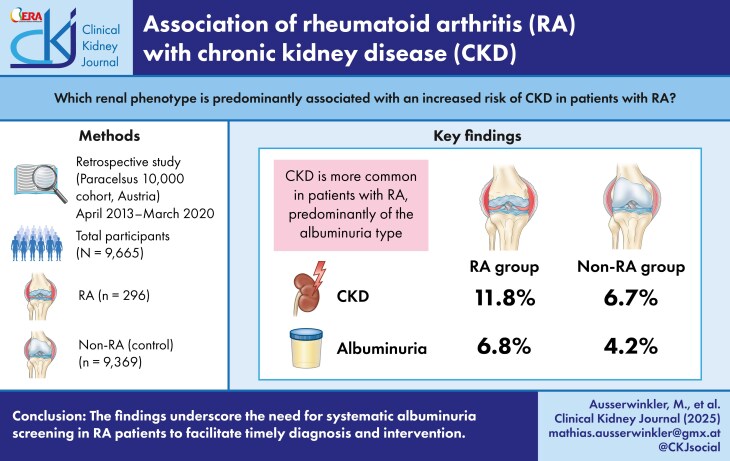

KEY LEARNING POINTS **What was known:**

- Rheumatoid arthritis (RA) has been associated with an increased risk of chronic kidney disease (CKD), but the predominant renal phenotype in RA remained unclear.
- Prior studies suggested that systemic inflammation and comorbidities may contribute to kidney impairment in RA.
- It was uncertain whether RA independently increases CKD risk after accounting for cardiovascular and metabolic factors.

**This study adds:**

- RA patients in a population-based cohort exhibited a distinct renal phenotype characterized predominantly by albuminuria at preserved estimated glomerular filtration rate (eGFR) rather than reduced eGFR.
- The RA–CKD association was substantially attenuated after adjustment for metabolic comorbidities, suggesting these factors largely mediate the relationship.
- A significant interaction with non-steroidal anti-inflammatory drugs (NSAIDs) use showed the RA–CKD association was confined to individuals not regularly using NSAIDs, reflecting confounding by indication rather than a protective effect.

**Potential impact:**

- Systematic albuminuria screening in RA patients may enable earlier identification of subclinical renal injury that would be missed by eGFR alone.
- Integrating nephrology-focused monitoring into rheumatology care may improve prevention of long-term renal and cardiovascular complications.
- Awareness of metabolic risk factor burden in RA may guide targeted interventions to mitigate CKD development.

## INTRODUCTION

Chronic kidney disease (CKD) and rheumatoid arthritis (RA) are highly prevalent conditions that contribute substantially to the global burden of disease [1–4]. CKD affects approximately 10% of the adult population and is a major driver of cardiovascular events, reduced quality of life and healthcare costs [5]. RA is a chronic autoimmune disease characterized by systemic inflammation and a high burden of comorbidity, including stroke, ischemic heart disease and kidney complications [6–8].

Growing evidence suggests that RA is independently associated with an increased risk of CKD and that higher RA disease activity is associated with accelerated eGFR decline and increased risk of clinically relevant kidney dysfunction [9]. Large population-based cohort studies have consistently demonstrated that RA patients are more likely to develop CKD compared with the general population, even after adjustment for established risk factors such as hypertension, diabetes and dyslipidemia. For instance, a nationwide cohort analysis reported a 31% increased risk of CKD among RA patients. Furthermore, Mendelian randomization data support a direct causal relationship, indicating that genetically determined RA increases the risk of CKD, kidney failure and related complications [10].

Mechanistically, the association is likely multifactorial. Chronic systemic inflammation, central to RA pathogenesis, is known to cause endothelial dysfunction, glomerular injury and kidney fibrosis [11]. Elevated high-sensitivity C-reactive protein (hsCRP) levels and high RA disease activity have been independently linked to accelerated kidney function decline. Additionally, extra-articular kidney disease (e.g. glomerulonephritis, amyloidosis) and potentially nephrotoxic medications such as non-steroidal anti-inflammatory drugs (NSAIDs) or certain disease-modifying agents contribute to development of CKD in RA [9, 12]. On the other hand, biologic agents that reduce systemic inflammation may attenuate this risk [13].

However, the extent to which RA increases CKD risk independently of established risk factors, systemic inflammation and medication exposures remains uncertain. Moreover, a paucity of data exists on potential interactions between RA and the above-mentioned risk factors, modification of the risk by inflammation levels or medication exposures. Using a large population-based cohort with comprehensive metabolic and inflammatory profiling, we examined whether the RA-CKD association is mediated by systemic inflammation and modified by antirheumatic therapy. Specifically, we investigated potential interactions between RA and traditional risk factors, inflammatory markers and medication exposures.

## MATERIALS AND METHODS

### Study population

This retrospective study used data from the Paracelsus 10,000 cohort, a population-based observational study conducted in Salzburg, Austria. The cohort consisted of participants aged 40–77 years who underwent baseline assessments between April 2013 and March 2020 [14]. Recruitment aimed to randomly select individuals from the Austrian national registry of residents, with approximately 56 600 invitation letters sent out, resulting in 10 044 participants being examined. For this analysis, we included 9665 participants with complete information on RA diagnosis and kidney function parameters. Individuals diagnosed with RA were identified based on American College of Rheumatology/European Alliance of Associations for Rheumatology (ACR/EULAR) classification criteria, requiring a total score of 6 or more out of 10 points across four key domains [15]. After this assessment detailed rheumatologic phenotyping beyond case classification was not available in the Paracelsus 10,000 dataset. In particular, we did not have information on RA disease duration, composite disease activity scores or serostatus (rheumatoid factor and anti-citrullinated protein antibodies) for each single case. We also did not capture exposure to disease-modifying antirheumatic drugs (DMARDs). The control group comprised individuals without RA from the cohort, excluding patients with chronic inflammatory bowel diseases and other rheumatic diseases [2, 14, 16, 17].

### Clinical assessments

All participants underwent standardized clinical, laboratory and imaging assessments. Blood samples were collected after an overnight fast for analysis of lipid profiles, glucose metabolism markers (fasting glucose and HbA1c), inflammatory markers (hsCRP) and kidney function parameters. Anthropometric measurements included body mass index and waist circumference. Blood pressure was measured bilaterally in a seated position, repeated three times per side after a 60-s resting interval. Participants completed structured interviews capturing personal medical history, medication use and lifestyle factors including smoking and alcohol consumption. The study collected data on medications addressing cardiovascular and metabolic risk factors, including antihypertensives, diabetes treatments, statins and NSAIDs.

### Kidney function assessment

CKD was defined according to KDIGO guidelines as an estimated glomerular filtration rate (eGFR) <60 mL/min/1.73 m² or albumin–creatinine ratio (ACR) ≥30 mg/g creatinine. eGFR was calculated using the Chronic Kidney Disease Epidemiology Collaboration 2009 equation based on serum creatinine levels. CKD staging was performed according to the KDIGO classification system combining CKD stages, eGFR categories (G1–G5) with albuminuria categories (A1–A3). Albuminuria was assessed using spot urine samples and categorized as A1 (ACR <30 mg/g), A2 (ACR 30–299 mg/g) or A3 (ACR ≥300 mg/g) [8].

### Statistical analysis

Continuous variables were expressed as median and interquartile range and compared using the Wilcoxon rank-sum test. Categorical data were expressed as percentages and compared using chi-squared tests. A two-sided significance level of *P* < .05 was used for all analyses.

The primary exposure was the diagnosis of RA. The primary endpoint was the presence of CKD (binary variable). Secondary endpoints included CKD stage and albuminuria categories. We fitted multiple logistic regression models: an unadjusted model, a model adjusted for Systematic COronary Risk Evaluation 2 (SCORE2) cardiovascular risk, and a comprehensive multivariable model adjusted for age categories, sex, metabolic syndrome, smoking status, NSAID use and hsCRP.

For albuminuria analysis, we used ordinal logistic regression to examine the association between RA and albuminuria categories. Interaction terms were tested individually between RA and all covariates to explore potential effect modification. All analyses were performed using Stata 18/BE.

### Ethics

The study was conducted in accordance with the Declaration of Helsinki and approved by the ethics committee of the federal state of Salzburg (415-E/1521/3–2012). All participants provided written informed consent.

## RESULTS

### Study population and baseline characteristics

A total of 9665 participants were included in the analysis, of whom 296 (3.1%) were diagnosed with RA. Participants with RA were significantly older than those without RA (median age 59 vs 55 years, *P* < .001) and more likely to be female (68% vs 51%, *P* < .001). RA patients had a higher prevalence of metabolic syndrome (35% vs 25%, *P* < .001) and were more likely to have abdominal obesity (58% vs 42%, *P* < .001), elevated blood pressure (66% vs 59%, *P* = .029) and elevated glucose levels (33% vs 27%, *P* = .023). Regular NSAID use was markedly higher in RA patients (21% vs 7%, *P* < .001). Cardiovascular risk profiles differed significantly, with RA patients showing higher SCORE2 risk scores (median 4.8 vs 4.0, *P* < .001) and elevated hsCRP levels (median 0.14 vs 0.12 mg/dL, *P* = .002; Table 1).

**Table 1:** Baseline characteristics of study participants with and without RA; data include demographic, metabolic and inflammatory parameters.

Characteristic	Total (*N* = 9665)	No RA (*N* = 9369)	RA (*N* = 296)	*P*-value
Demographics				
Age (years), median (IQR)	55 (50–62)	55 (50–62)	59 (54–64)	<.001
Age categories, *n* (%)				<0.001
<50 years	2335 (24.2)	2307 (24.6)	28 (9.5)	
50–59 years	4145 (42.9)	4015 (42.8)	130 (43.9)	
60+ years	3185 (33.0)	3047 (32.5)	138 (46.6)	
Male sex, *n* (%)	4684 (48.5)	4588 (49.0)	96 (32.4)	<.001
Anthropometric measures				
BMI (kg/m²), median (IQR)	26.0 (23.0–29.0)	26.0 (23.0–29.0)	27.0 (23.0–31.0)	<.001
BMI ≥30 kg/m², *n* (%)	1853 (19.2)	1753 (18.7)	100 (33.8)	<.001
Metabolic parameters				
Metabolic syndrome, *n* (%)	2454 (25.4)	2351 (25.1)	103 (34.8)	<.001
Abdominal obesity	4098 (42.4)	3925 (41.9)	173 (58.4)	<.001
Elevated triglycerides	2513 (26.0)	2429 (25.9)	84 (28.4)	.34
Low HDL cholesterol	1016 (10.5)	976 (10.4)	40 (13.5)	.084
Elevated blood pressure	5724 (59.2)	5530 (59.0)	194 (65.5)	.029
Elevated glucose	2607 (27.0)	2510 (26.8)	97 (32.8)	.023
Lifestyle factors				
Smoking status, *n* (%)				0.040
Never smoker	4106 (44.9)	3999 (45.4)	107 (38.1)	
Previous smoker	3369 (36.8)	3247 (36.9)	122 (43.4)	
Current smoker	1674 (18.3)	1623 (18.4)	51 (18.1)	
NSAID use, *n* (%)	728 (7.5)	667 (7.1)	61 (20.6)	<.001
Cardiovascular risk				
SCORE2 risk, median (IQR)	4.0 (2.1–6.8)	4.0 (2.1–6.8)	4.8 (2.6–7.8)	<.001
Laboratory parameters				
Creatinine (mg/dL), median (IQR)	0.8 (0.7–1.0)	0.8 (0.7–1.0)	0.8 (0.7–0.9)	<.001
eGFR (mL/min/1.73 m²), median (IQR)	89 (79–98)	89 (79–98)	90 (77–97)	.72
hsCRP (mg/dL), median (IQR)	0.12 (0.06–0.24)	0.12 (0.06–0.24)	0.14 (0.08–0.28)	.002
Medications				
Antihypertensive therapy, *n* (%)	1998 (20.7)	1901 (20.3)	97 (32.8)	<.001
Statin therapy, *n* (%)	799 (8.3)	767 (8.2)	32 (10.8)	.11

BMI, body mass index; HDL, high-density lipoprotein; IQR, interquartile range.

### Kidney disease outcomes

**Figure 1:**
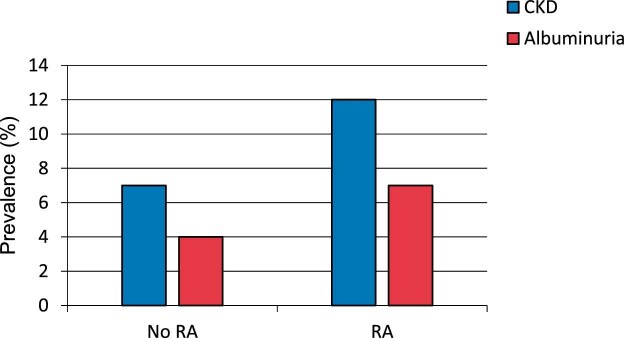
Distribution of ACR in participants with and without rheumatoid arthritis. RA patients exhibit a higher proportion of albuminuria even at preserved eGFR.

CKD was present in 664 participants (6.9%) overall, with a significantly higher prevalence in RA patients compared with controls (11.8% vs 6.7%, *P* < .001) (Table 2). The distribution of CKD stages revealed distinct patterns between groups (*P* = .006). Presence of albuminuria in the context of preserved eGFR was predominant in RA patients and controls (6.8% vs 4.2%). Additionally, RA patients showed an increased prevalence of higher GFR categories (G3a–G5: 4.7% vs 2.3%). The ACR was higher in RA patients (median 4 vs 3 mg/g, *P* = .002), with 7.7% having ACR ≥30 mg/g compared with 4.8% in controls (*P* = .028, Fig. 1).

**Table 2:** Multivariable ORs with 95% CIs and interaction effects with RA for various demographic characteristics, metabolic syndrome components, lifestyle factors, NSAID use and hsCRP.

Variable	Multivariable, OR (95% CI)	*P*-value	Interaction with RA, OR (95% CI)	*P*-value
RA	1.43 (0.96–2.13)	.079		
Age categories				
<50 years	Reference		Reference	
50–59 years	1.15 (0.88–1.52)	.304	2.11 (0.26–17.19)	.487
60+ years	2.78 (2.14–3.61)	<.001	1.40 (0.18–10.97)	.751
Male sex	0.90 (0.76–1.07)	.249	1.64 (0.79–3.44)	.186
Metabolic syndrome	2.00 (1.67–2.40)	<.001	0.88 (0.42–1.85)	.740
Metabolic syndrome components				
Abdominal obesity			0.64 (0.30–1.37)	.252
Elevated triglycerides			1.22 (0.58–2.56)	.596
Low HDL cholesterol			0.59 (0.21–1.65)	.314
Hypertension			1.70 (0.63–4.59)	.299
Elevated glucose			1.00 (0.48–2.08)	.999
Smoking status				
Never smoker	Reference		Reference	
Previous smoker	0.88 (0.73–1.07)	.208	1.17 (0.50–2.73)	.715
Current smoker	1.16 (0.92–1.47)	.218	1.23 (0.44–3.48)	.693
NSAID use	1.51 (1.16–1.96)	.002	0.40 (0.17–0.97)	.042
hsCRP (per unit)	1.35 (1.19–1.54)	<.001	0.99 (0.64–1.53)	.955

HDL, high-density lipoprotein.

### Association between rheumatoid arthritis and chronic kidney disease

In unadjusted analysis RA was significantly associated with CKD [odds ratio (OR) 1.86, 95% confidence interval (CI) 1.30–2.68, *P* = .001]. This association remained virtually unchanged after adjustment for cardiovascular risk using the SCORE2 calculator (OR 1.86, 95% CI 1.27–2.73, *P* = .001), suggesting that cardiovascular risk factors do not explain the observed relationship.

However, comprehensive multivariable adjustment substantially attenuated the association. After controlling for age categories, sex, metabolic syndrome, smoking status, NSAID use and hsCRP, the association between RA and CKD became non-significant (OR 1.43, 95% CI 0.96–2.13, *P* = .079), representing a 23% reduction in the odds ratio compared with the unadjusted analysis (Table 3).

**Table 3:** Prevalence and distribution of CKD stages in participants with and without RA; classification based on eGFR and albuminuria.

Outcome	Total (*N* = 9665)	No RA (*N* = 9369)	RA (*N* = 296)	*P*-value
CKD				
CKD (binary), *n* (%)	664 (6.9)	629 (6.7)	35 (11.8)	<.001
CKD stages (GFR + ACR categories), *n* (%)				0.006
G1A1 (eGFR ≥90, ACR <30)	4319 (44.7)	4182 (44.6)	137 (46.3)	
G1A2 (eGFR ≥90, ACR 30–299)	205 (2.1)	194 (2.1)	11 (3.7)	
G1A3 (eGFR ≥90, ACR ≥300)	11 (0.1)	10 (0.1)	1 (0.3)	
G2A1 (eGFR 60–89, ACR <30)	4682 (48.4)	4558 (48.6)	124 (41.9)	
G2A2 (eGFR 60–89, ACR 30–299)	208 (2.2)	199 (2.1)	9 (3.0)	
G2A3 (eGFR 60–89, ACR ≥300)	15 (0.2)	14 (0.1)	1 (0.3)	
G3aA1 (eGFR 45–59, ACR <30)	176 (1.8)	166 (1.8)	10 (3.4)	
G3aA2 (eGFR 45–59, ACR 30–299)	22 (0.2)	22 (0.2)	0 (0.0)	
G3aA3 (eGFR 45–59, ACR ≥300)	2 (0.0)	2 (0.0)	0 (0.0)	
G3bA1 (eGFR 30–44, ACR <30)	9 (0.1)	8 (0.1)	1 (0.3)	
G3bA2 (eGFR 30–44, ACR 30–299)	5 (0.1)	5 (0.1)	0 (0.0)	
G3bA3 (eGFR 30–44, ACR ≥300)	1 (0.0)	1 (0.0)	0 (0.0)	
G4A1 (eGFR 15–29, ACR <30)	3 (0.0)	2 (0.0)	1 (0.3)	
G4A2 (eGFR 15–29, ACR 30–299)	2 (0.0)	2 (0.0)	0 (0.0)	
G4A3 (eGFR 15–29, ACR ≥300)	4 (0.0)	3 (0.0)	1 (0.3)	
G5A3 (eGFR <15, ACR ≥300)	1 (0.0)	1 (0.0)	0 (0.0)	
ACR				
ACR (mg/g), median (IQR)	3 (2–7)	3 (2–7)	4 (2–9)	.002
ACR categories, *n* (%)				0.028
ACR <30	9201 (95.1)	8927 (95.2)	274 (92.3)	
ACR 30–300	443 (4.6)	423 (4.5)	20 (6.7)	
ACR >300	34 (0.4)	31 (0.3)	3 (1.0)	
Summary patterns				
Normal kidney function (G1A1 + G2A1)	9001 (93.1)	8740 (93.3)	261 (88.2)	<.001
Albuminuria at preserved GFR (G1A2 + G2A2)	413 (4.3)	393 (4.2)	20 (6.8)	.027
Reduced GFR (G3a–G5)	225 (2.3)	211 (2.3)	14 (4.7)	.005

### Interaction analysis

To explore potential effect modification, we tested interactions between RA and all covariates in the multivariable model. A significant interaction was observed between RA and NSAID use (*P* = .042), suggesting that the association between RA and CKD varies by NSAID use status. Among participants not using NSAIDs, RA remained significantly associated with CKD (OR 2.02, 95% CI 1.34–3.05, *P* = .001), while the interaction term indicated a modified effect in RA patients using NSAIDs (interaction OR 0.40, 95% CI 0.17–0.97, *P* = .042).

No significant interactions were found between RA and age categories (50–59 years: interaction OR 2.11, 95% CI 0.26–17.19, *P* = .487; 60+ years: interaction OR 1.40, 95% CI 0.18–10.97, *P* = .751), sex (interaction OR 1.64, 95% CI 0.79–3.44, *P* = .186), metabolic syndrome (interaction OR 0.88, 95% CI 0.42–1.85, *P* = .740), smoking status (previous smoking: interaction OR 1.17, 95% CI 0.50–2.73, *P* = .715; current smoking: interaction OR 1.23, 95% CI 0.44–3.48, *P* = .693) or individual metabolic syndrome components (all *P* > .05). Similarly, no interaction was observed between RA and hsCRP levels (interaction OR 0.99, 95% CI 0.64–1.53, *P* = .955).

### Albuminuria-specific analysis

Given the observed pattern of increased albuminuria in RA patients, we performed ordinal logistic regression to examine the association between RA and albumin–creatinine ratio categories. In unadjusted analysis, RA patients had significantly higher odds of more severe albuminuria categories (OR 1.66, 95% CI 1.07–2.56, *P* = .023). This association became slightly stronger after adjustment for cardiovascular risk using SCORE2 (OR 1.72, 95% CI 1.09–2.71, *P* = .019), suggesting that traditional cardiovascular risk factors may have been acting as negative confounders.

However, after comprehensive multivariable adjustment for age categories, sex, metabolic syndrome, smoking status, NSAID use and hsCRP, the association between RA and albuminuria severity was no longer statistically significant (OR 1.43, 95% CI 0.90–2.28, *P* = .132). This pattern paralleled the findings for binary CKD outcomes, suggesting that the relationship between RA and kidney disease outcomes is mediated through similar pathways regardless of the specific outcome measure.

## DISCUSSION

In this large, population-based cohort, we found that RA was associated with a higher prevalence of CKD. Importantly, the predominant renal manifestation was albuminuria with preserved eGFR, rather than reduced eGFR. This phenotype suggests that early glomerular involvement is common in RA and highlights the need to assess both ACR and eGFR when evaluating kidney health in this population. Our findings extend previous research linking RA to an increased risk of CKD. Large cohort studies have consistently shown that RA patients are more likely to develop CKD compared with the general population [9]. Mendelian randomization analyses also support a causal effect of RA on kidney outcomes [11]. Earlier studies emphasized eGFR decline as the key feature of RA-related kidney disease [13]. By contrast, our data show that albuminuria is more frequent than reduced eGFR in RA patients, indicating early, subclinical renal injury. Mechanistically, this may reflect immune-mediated endothelial dysfunction, microvascular injury or subclinical glomerulonephritis [18, 19]. Importantly, because detailed rheumatology-specific data such as disease duration, serostatus and antirheumatic treatment exposure were not available, we cannot determine which RA-related factors (autoantibody-positive disease, chronic inflammatory activity or DMARD exposure) are driving the observed renal phenotype. Our findings therefore describe the renal phenotype of RA at a population level but do not allow causal or mechanistic inference.

The association between RA and CKD was attenuated after multivariable adjustment, suggesting that much of the excess risk is mediated by comorbidities such as obesity, hypertension and diabetes, which are highly prevalent in RA [20–22]. Nevertheless, the persistence of albuminuria in RA patients points to an additional disease-specific contribution beyond traditional risk factors. We also observed that the association between RA and CKD was confined to participants not reporting regular NSAID use. This counterintuitive finding is best explained by confounding by indication or reverse causation: patients at higher renal risk may avoid NSAIDs, while regular users may represent a healthier subset with preserved kidney function. Prior evidence shows that long-term NSAID use increases CKD risk, and prescribing patterns often reflect disease severity and baseline renal status [23, 24]. Thus, our results should not be interpreted as evidence of a protective effect of NSAIDs.

Strengths of this study include the large, unselected population sample, detailed metabolic and laboratory profiling, and the combined assessment of eGFR and albuminuria. This study has limitations. First, its cross-sectional design precludes causal inference regarding the direction of the association between RA and kidney injury. Second, although RA was defined according to established ACR/EULAR criteria, we lacked key rheumatology-specific variables, including disease duration, composite disease activity measures, serostatus and current or cumulative exposure to DMARDs. Third, albuminuria was assessed from a single spot urine sample, which may overestimate persistent proteinuria [13, 25].

In conclusion, albuminuria with preserved eGFR emerges as the predominant renal phenotype in RA, whereas reduced eGFR is less common. These findings emphasize the importance of routine albuminuria screening in RA patients, complementing eGFR measurement, to enable earlier detection and timely intervention. Multidisciplinary care models integrating nephrology into rheumatology practice may help improve long-term renal and cardiovascular outcomes.

## Data Availability

The data underlying this article are available in the article.
